# Comparative effects of the Mandibular Protraction Appliance in adolescents and adults

**DOI:** 10.1590/2177-6709.23.3.063-072.oar

**Published:** 2018

**Authors:** Bruno D’Aurea Furquim, Guilherme Janson, Laura de Castro Cabrera Cope, Karina Maria Salvatore Freitas, José Fernando Castanha Henriques

**Affiliations:** 1 Private practice (Maringá/PR, Brazil).; 2 Universidade de São Paulo, Faculdade de Odontologia de Bauru, Departamento de Ortodontia (Bauru/SP, Brazil).; 3 Independent researcher currently not affiliated (Dallas/TX, USA).; 4 Centro Universitário Ingá Uningá, Departamento de Ortodontia (Maringá/PR, Brazil).

**Keywords:** Orthodontics, Functional orthodontic appliances, Corrective orthodontics

## Abstract

**Objective:**

The aim of this study was to compare the skeletal, dental, and soft tissue effects of the Mandibular Protraction Appliance (MPA) application in adolescent and adult Class II malocclusion patients.

**Methods:**

The sample comprised the pretreatment and posttreatment lateral cephalograms of 39 subjects presenting Class II malocclusion treated with the MPA and fixed appliances. Sample was divided into two groups: Group 1 comprised 23 subjects (10 male; 13 female), at a mean pretreatment age of 11.75 years, with a mean treatment time of 3.32 years; Group 2 included 16 subjects (7 male; 9 female), at a mean pretreatment age of 22.41 years, with a mean treatment time of 4.24 years. Intergroup comparison of the initial and final stages and treatment changes between the groups was performed with *t* tests, at *p*< 0.05.

**Results:**

The adults showed less significant amounts of skeletal, dentoalveolar and soft tissue changes than the adolescents. There was significantly greater palatal tipping of the maxillary incisors and retrusion of the upper lip in the adolescents. The adult group showed greater mandibular incisor proclination in the posttreatment stage.

**Conclusion:**

Adult patients treated with MPA showed less significant amounts of skeletal, dentoalveolar and soft tissue changes than adolescents.

## RESUMO

**Objetivo:**

o objetivo do presente estudo foi comparar os efeitos esqueléticos, dentários e em tecidos moles do Aparelho de Protração Mandibular (APM) em pacientes adolescentes e adultos com má oclusão de Classe II.

**Métodos:**

a amostra consistiu das telerradiografias pré- e pós-tratamento de 39 indivíduos com má oclusão de Classe II tratados com APM e aparelhos fixos. A amostra foi dividida em dois grupos: o Grupo 1 compreendeu 23 indivíduos (10 homens e 13 mulheres), com idade inicial de 11,75 anos, com um tempo médio de tratamento de 3,32 anos; o Grupo 2 incluiu 16 pacientes (7 do sexo masculino e 9 do sexo feminino), com idade média inicial de 22,41 anos, com um tempo médio de tratamento de 4,24 anos. A comparação intergrupos dos estágios inicial e final e das alterações com o tratamento foi realizada com testes *t*, considerando-se um nível de significância de *p*< 0,05.

**Resultados:**

os adultos apresentaram quantidades menos significativas de alterações esqueléticas, dentoalveolares e de tecidos moles do que os adolescentes. Houve uma inclinação palatina significativamente maior dos incisivos superiores e retrusão do lábio superior nos adolescentes. O grupo de adultos apresentou maior proclinação dos incisivos inferiores no estágio pós-tratamento.

**Conclusão:**

pacientes adultos tratados com APM apresentaram quantidades menos significativas de alterações esqueléticas, dentoalveolares e de tecidos moles do que os adolescentes.

## INTRODUCTION AND STATEMENT OF THE PROBLEM

The number of adults seeking orthodontic treatment is rising; however,^1^ ^,^ ^2^ because there is no growth potential, certain conditions cannot be resolved with braces alone. Sometimes, surgery and extractions are required in order to obtain the proper results.^3^ ^-^ ^8^

The interest in functional appliances to correct anteroposterior jaw discrepancies has been emphasized over the past two decades.^7^ ^,^ ^9^ ^-^ ^11^ Nevertheless, studies have indicated that a great amount of the corrections seem to occur through dentoalveolar movements rather than through skeletal changes, stimulating some clinicians to use them in nongrowing patients.^12^

The Mandibular Protraction Appliance (MPA), developed by Coelho Filho^13^ in 1995, is a handmade functional appliance to correct Class II malocclusions. It functions much like the Herbst appliance,^14^ but has a smaller design and is attached to the maxillary first molar headgear tube and to the mandibular rectangular archwire. The MPA is an inexpensive, simple and effective appliance in adolescents.^15^

Some clinical reports and studies of nongrowing patients treated with the MPA have been described in the literature,^11^ ^,^ ^16^ and one study compared treatment changes of children, adolescents and adult patients, using the MPA.^9^ Pontes et al^9^ found no difference in anteroposterior correction among the three evaluated groups. With the objective of clarifying this matter, the present study aimed to compare the skeletal, dental, and soft tissue effects of the Mandibular Protraction Appliance (MPA) associated to fixed appliances in adolescent and adult Class II malocclusion subjects. 

## MATERIAL AND METHODS

### Material

The sample size of each group was calculated based on an alpha significance level of 0.05 and a beta of 0.2 to achieve 80% of power to detect a mean difference of 0.5^o^ in ANB angle change between the groups, with 0.5^o^ of estimated standard deviation.^17^ The sample size calculation showed that 9 patients in each group were needed.

The sample comprising this retrospective study consisted of the pretreatment and posttreatment lateral cephalograms of 39 patients presenting with Class II malocclusion treated at two different orthodontic private practices. Patients presented mild to moderate Class II malocclusion. All subjects were in the permanent dentition up to first molars erupted when MPA was installed. No exclusion criteria related to occlusal result was adopted.

**Figure 1:**
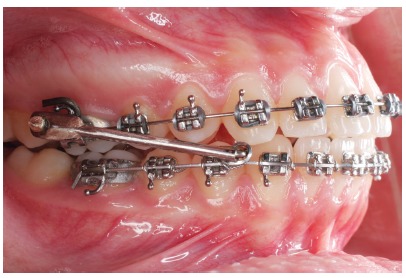
The Mandibular Protraction Appliance.

The sample was divided into two groups:

» Group 1 comprised 23 subjects (10 male; 13 female), at a mean pretreatment age of 11.75 ± 1.13 years, treated with the Mandibular Protraction Appliance and fixed appliances (Fig 1), for a mean treatment time of 3.32 ± 1.20 years, at two orthodontic private practices. Class II division 1 was present in 21 subjects and division 2, in 2 patients. Nineteen had bilateral Class II and 4 showed Class II subdivision malocclusion.
» Group 2 comprised 16 subjects (7 male; 9 female), at a mean pretreatment age of 22.41 ± 4.79 years, also treated with the Mandibular Protraction Appliance and fixed appliances (Fig 1), for a mean treatment time of 4.24 ± 2.44 years at two orthodontic private practices. Twelve subjects presented Class II division 1 malocclusion and 4 had Class II division 2. Class II subdivision was seen in 5 subjects and bilateral Class II was present in 11 patients.

The mean MPA treatment time was 9 months in both groups. After removal of the MPA, active retention was provided with Class II elastics to maintain the anteroposterior relationship correction. Gradual decrease in Class II elastics use was conducted in each case, as stability of the anteroposterior relationship was observed.

### Methods

Lateral cephalograms were obtained at the pre (T_1_) and posttreatment (T_2_) stages. The anatomic tracing and the location of cephalometric landmarks were manually carried out by a single investigator. These radiographs were digitized in grayscale at 300 dpi, in a scanner (Numonics AccuGrid XNT, model A30TL.F (Austin,Texas/USA) and imported into Dentofacial Planner 7.02 software (Toronto, Ontário, Canada); the landmarks were digitized and measurements were performed. The magnification factor of the radiographic images, which was between 6% and 9.8%, was corrected by the software.

**Figure 2:**
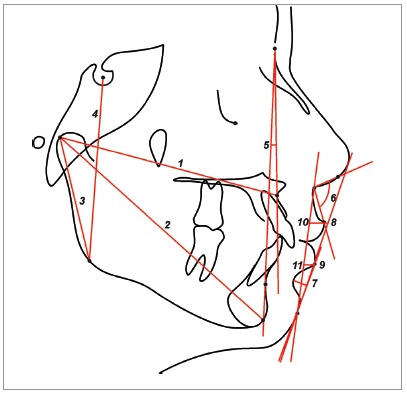
Skeletal and soft tissue variables: 1) Co-A; 2) Co-Gn; 3) Co-Go; 4) S-Go; 5) NAP; 6) NLA; 7) MLS; 8) UL-E; 9) LL-E; 10) UL-Pog’Sn; 11) LL-Pog’Sn.

**Figure 3:**
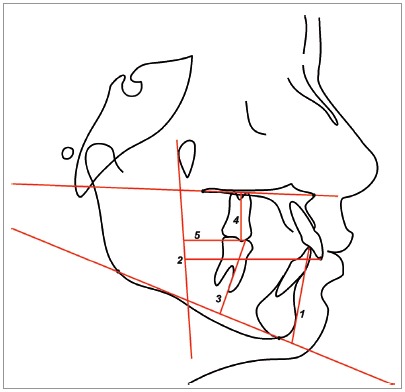
Dentoalveolar variables 1) 1-PM; 2) 1-PTV; 3) 6-PM; 4) 6-PP; 5) 6-PTV.

Cephalometric maxillary and mandibular dentoalveolar variables used are shown in Figures 2 and 3.

### Error study

Within a month interval from the first measurement, 15 randomly selected radiographs were re-measured by the same examiner. The random error was calculated according to Dahlberg’s formula (Se^2^= Σd^2^/2n),^18^ in which Se^2^ is the error variance and *d* is the difference between two determinations of the same variable. The systematic errors were evaluated with dependent *t* tests for *p*< 0.05.^19^

### Statistical analysis

Sex distribution, type of Class II malocclusion (division 1 or 2; bilateral or unilateral) and number of patients treated by the different clinicians in the groups were compared with Chi-square tests. Application of *t* tests requires normal distribution, which was verified with Kolmogorov-Smirnov tests. Results were not significant for all variables. Therefore, intergroup comparison of the pretreatment age, treatment time, pretreatment (T_1_) and posttreatment (T_2_) variables and the treatment changes (T_2_-T_1_) were performed with *t* tests. 

All statistical analyses were performed with Statistica software (Statistica for Windows 6.0; Statsoft, Tulsa, Okla), and results were considered significant at *p*< 0.05.

## RESULTS

Only the variables 1-PTV and 1-PP showed systematic errors, and the random errors ranged from 0.28 millimeters (MLS) to 2.80 degrees (1.PP). 

The groups were comparable regarding sex distribution, types of Class II malocclusion and also regarding the number of patients treated by both clinicians (Table 1). Only the pre and posttreatment ages were significantly different between the groups (Table 2).

**Table 1:** Distribution between the two groups regarding sex, malocclusion type (divisions 1 and 2; bilateral or unilateral) and orthodontist (Chi-square tests).

Group / Sex	Female	Male	Total
1	13	10	23
2	9	7	16
TOTAL	22	17	39
X^2^ = 0.00 DF= 1 p = 0.986			
Group / Division	Division 1	Division 2	Total
1	21	2	23
2	12	4	16
TOTAL	33	6	39
X^2^ = 1.93 DF= 1 p = 0.165			
Group Subdivision	Bilateral	Subdivision	Total
1	19	4	23
2	11	5	16
TOTAL	30	9	39
X^2^ = 1.02 DF= 1 p = 0.312			
Group / Orthodontist	Orthodontist 1	Orthodontist 2	Total
1	16	7	23
2	11	5	16
TOTAL	27	12	39
X^2^ = 0.00 DF= 1 p = 0.957			

**Table 2:** Intergroup comparison of pretreatment stage

VARIABLES	GROUP 1 (n = 23)		GROUP 2 (n = 16)		p
	Mean	SD	Mean	SD	
Chronological age					
Age (years)	11.75	1.13	22.41	4.79	0.000*
Maxillary skeletal component					
SNA (degrees)	82.84	3.92	80.96	5.60	0.223
A-Nperp (mm)	2.19	2.56	-0.28	2.98	0.009*
Co-A (mm)	84.72	4.60	86.34	6.25	0.356
Mandibular skeletal component					
SNB (degrees)	77.44	3.23	77.36	5.31	0.956
Pog-Nperp (mm)	-3.50	4.60	-3.36	4.95	0.929
Co-Gn (mm)	104.37	8.16	110.20	7.60	0.030*
Go-Gn (mm)	70.11	5.37	74.55	4.63	0.011*
Co-Go (mm)	47.28	4.80	52.23	6.18	0.008*
Maxillomandibular relationship					
ANB (degrees)	5.38	2.11	3.59	2.40	0.018*
NAP (degrees)	9.30	5.37	3.17	5.96	0.002*
Vertical component					
FMA (degrees)	25.24	5.61	22.69	7.28	0.223
SN.GoGn (degrees)	32.42	4.56	29.05	9.38	0.143
SN.PP (degrees)	7.41	4.14	7.35	4.64	0.967
LAFH (mm)	60.22	7.50	63.08	5.87	0.210
S-Go (mm)	66.56	6.19	74.52	7.79	0.001*
Maxillary dentoalveolar component					
1.PP (degrees)	115.90	7.63	111.58	12.22	0.182
1-PP (mm)	26.13	3.48	26.83	2.75	0.507
1.NA (degrees)	25.67	8.14	23.30	13.53	0.500
1-NA (mm)	4.27	2.94	4.86	4.65	0.631
1-PTV (mm)	56.29	4.07	56.47	4.94	0.901
6-PP (mm)	19.86	2.75	22.84	2.39	0.001*
6-PTV (mm)	23.09	3.69	26.83	3.36	0.003*
Mandibular dentoalveolar component					
IMPA (degrees)	96.27	7.06	99.54	5.99	0.139
1.NB (degrees)	28.50	7.21	28.38	6.24	0.959
1-NB (mm)	4.54	2.37	4.29	2.72	0.756
1-MP (mm)	37.39	3.88	38.80	3.50	0.254
1-PTV (mm)	49.82	4.96	51.24	5.31	0.400
6-MP (mm)	26.29	3.26	28.57	2.89	0.030*
6-PTV (mm)	22.49	3.83	26.88	3.96	0.001*
Dental relationships					
Overjet (mm)	6.47	2.61	5.23	2.70	0.161
Overbite (mm)	4.16	1.80	3.45	2.09	0.266
Molar relationship (mm)	0.60	1.55	-0.04	1.88	0.250
Soft tissue component					
NLA (degrees)	105.98	14.09	110.51	11.43	0.295
MLS (mm)	5.08	1.37	6.28	1.19	0.007*
UL-E (mm)	-1.46	1.47	-4.22	2.18	0.000*
LL-E (mm)	-0.52	2.21	-2.84	2.71	0.006*
UL-Pog’Sn (mm)	4.26	1.44	2.49	1.81	0.002*
LL-Pog’Sn (mm)	2.00	2.07	0.68	2.80	0.098

* Statistically significant for *p* < 0.05.

At the pretreatment stage, Group 2 presented significantly greater maxillary retrusion (A-Nperp), mandibular effective and body length (Co-Gn, Go-Gn), and mandibular ramus (Co-Go), when compared to Group 1 (Table 2). Group 1 had a significantly greater skeletal Class II discrepancy (ANB, and facial convexity, NAP), and a significantly smaller posterior face height (S-Go) than Group 2. Group 2 also presented greater maxillary and mandibular molar extrusion (6-PP, 6-PM) and mesialization (6-PTV, 6-PTV), and showed deeper mentolabial sulcus (MLS) and greater upper (UL-E, UL-Pog’Sn) and lower (LL-E) lip retraction.

At the posttreatment stage, Group 2 showed significantly greater maxillary retrusion (A-Nperp), smaller apical base discrepancy (ANB) and less convex facial profile (NAP) than Group 1 (Table 3). Group 2 also presented greater maxillary incisors labial inclination and protrusion (1.NA, 1-NA), greater mandibular incisors labial inclination (IMPA), smaller overbite, deeper mentolabial sulcus (MLS) and greater retrusion of the upper lip (UL-E), in relation to Group 1. 

**Table 3:** Intergroup comparison of posttreatment stage

VARIABLES	GROUP 1 (n = 23)		GROUP 2 (n = 16)		p
	Mean	SD	Mean	SD	
Chronological age					
Age (years)	15.07	1.53	26.65	5.88	0.000*
Maxillary skeletal component					
SNA (degrees)	82.80	3.71	81.09	6.05	0.274
A-Nperp (mm)	2.03	2.41	-0.07	3.43	0.029*
Co-A (mm)	88.19	5.26	86.19	6.09	0.277
Mandibular skeletal component					
SNB (degrees)	78.34	3.13	78.04	5.14	0.817
Pog-Nperp (mm)	-2.32	5.16	-1.94	4.85	0.815
Co-Gn (mm)	111.59	7.76	110.72	7.02	0.720
Go-Gn (mm)	74.28	5.02	74.45	4.76	0.913
Co-Go (mm)	52.28	5.33	53.09	5.52	0.644
Maxillomandibular relationship					
ANB (degrees)	4.46	1.68	3.06	2.57	0.044*
NAP (degrees)	6.89	4.59	2.08	6.40	0.009*
Vertical component					
FMA (degrees)	24.39	6.39	22.11	7.07	0.296
SN.GoGn (degrees)	31.39	5.84	28.39	8.93	0.207
SN.PP (degrees)	7.66	3.81	6.81	4.75	0.537
LAFH (mm)	64.05	7.83	64.34	5.37	0.900
S-Go (mm)	72.51	7.04	76.16	7.45	0.125
Maxillary dentoalveolar component					
1.PP (degrees)	108.74	5.72	112.16	5.80	0.073
1-PP (mm)	27.28	3.79	27.81	2.72	0.633
1.NA (degrees)	18.27	5.54	24.25	7.84	0.007*
1-NA (mm)	2.29	2.19	4.43	3.11	0.015*
1-PTV (mm)	55.54	4.51	55.94	4.70	0.791
6-PP (mm)	21.97	3.14	23.23	2.10	0.167
6-PTV (mm)	24.94	3.80	26.94	4.05	0.119
Mandibular dentoalveolar component					
IMPA (degrees)	100.21	5.80	105.19	6.28	0.014*
1.NB (degrees)	32.22	3.70	34.20	4.49	0.136
1-NB (mm)	5.61	2.04	5.55	1.91	0.928
1-MP (mm)	38.41	4.74	37.70	3.16	0.603
1-PTV (mm)	52.88	4.44	53.00	4.57	0.932
6-MP (mm)	29.98	2.96	30.44	2.54	0.618
6-PTV (mm)	27.29	3.86	29.01	4.10	0.187
Dental relationships					
Overjet (mm)	2.67	0.40	2.94	0.75	0.147
Overbite (mm)	2.25	0.84	1.38	1.37	0.017*
Molar relationship (mm)	-2.35	0.62	-2.06	0.51	0.127
Soft tissue component					
NLA (degrees)	109.91	12.42	111.53	11.47	0.680
MLS (mm)	4.57	1.43	5.81	1.33	0.009*
UL-E (mm)	-4.02	1.96	-5.46	2.16	0.034*
LL-E (mm)	-1.62	2.51	-3.09	2.25	0.065
UL-Pog’Sn (mm)	2.83	2.01	1.98	1.81	0.177
LL-Pog’Sn (mm)	1.64	2.41	0.91	2.05	0.330

* Statistically significant for p < 0.05.

The intergroup comparison of treatment changes showed that Group 1 presented significantly greater increase of maxillary (Co-A) and mandibular lengths (Co-Gn, Go-Gn, Co-Go) (Table 4). Group 1 also showed greater increase in lower anterior and posterior face heights (LAFH, S-Go), greater palatal tipping of the maxillary incisors (1.PP, 1.NA), greater maxillary molar extrusion and mesialization (6-PP, 6-PTV), greater mandibular incisor and molar extrusion (1-MP, 6-MP), greater mandibular molar mesialization (6-PTV) and greater upper lip retraction (UL-E), as compared to Group 2.

**Table 4:** Intergroup comparison of treatment changes.

VARIABLES	GROUP 1 (n = 23)		GROUP 2 (n = 16)		p
	Mean	SD	Mean	SD	
Treatment time					
Treat. time (years)	3.32	1.20	4.24	2.44	0.126
Maxillary skeletal component					
SNA (degrees)	-0.19	1.54	0.14	1.51	0.517
A-Nperp (mm)	-0.36	1.21	0.21	1.64	0.225
Co-A (mm)	3.02	2.11	-0.14	1.35	0.000*
Mandibular skeletal component					
SNB (degrees)	0.80	1.57	0.68	1.19	0.797
Pog-Nperp (mm)	0.89	2.02	1.42	2.71	0.490
Co-Gn (mm)	7.07	3.43	0.52	1.44	0.000*
Go-Gn (mm)	4.05	2.53	0.10	1.32	0.000*
Co-Go (mm)	4.73	2.81	0.86	1.83	0.000*
Maxillomandibular relationship					
ANB (degrees)	-0.97	1.56	-0.53	1.60	0.398
NAP (degrees)	-2.55	3.68	-1.09	3.48	0.221
Vertical component					
FMA (degrees)	-0.31	1.86	-0.58	1.63	0.639
SN.GoGn (degrees)	-0.54	2.33	-0.66	1.56	0.862
SN.PP (degrees)	0.28	1.76	-0.54	1.40	0.129
LAFH (mm)	4.11	3.10	1.26	1.64	0.002*
S-Go (mm)	5.80	2.85	1.64	1.67	0.000*
Maxillary dentoalveolar component					
1.PP (degrees)	-7.10	7.87	0.58	9.72	0.010*
1-PP (mm)	1.31	1.60	0.99	1.45	0.526
1.NA (degrees)	-7.22	7.76	0.95	10.51	0.008*
1-NA (mm)	-1.86	2.56	-0.44	3.61	0.158
1-PTV (mm)	-0.95	2.95	-0.53	2.48	0.643
6-PP (mm)	2.17	2.01	0.39	0.93	0.002*
6-PTV (mm)	1.65	1.86	0.11	2.88	0.050*
Mandibular dentoalveolar component					
IMPA (degrees)	3.64	6.87	5.64	4.96	0.325
1.NB (degrees)	3.81	6.55	5.82	5.06	0.310
1-NB (mm)	1.13	1.25	1.26	1.79	0.794
1-MP (mm)	1.14	2.58	-1.10	1.65	0.004*
1-PTV (mm)	2.83	2.38	1.76	2.33	0.172
6-MP (mm)	3.70	1.40	1.87	1.56	0.000*
6-PTV (mm)	4.54	2.08	2.13	3.09	0.006*
Dental relationships					
Overjet (mm)	-3.79	2.58	-2.29	2.62	0.086
Overbite (mm)	-1.90	1.60	-2.08	1.60	0.733
Molar relationship (mm)	-2.89	1.74	-2.02	2.08	0.166
Soft tissue component					
NLA (degrees)	3.01	12.25	1.03	5.74	0.551
MLS (mm)	-0.44	1.40	-0.48	1.11	0.941
UL-E (mm)	-2.51	2.01	-1.24	0.79	0.022*
LL-E (mm)	-0.96	1.23	-0.25	1.39	0.103
UL-Pog’Sn (mm)	-1.30	1.88	-0.52	0.84	0.130
LL-Pog’Sn (mm)	-0.17	0.99	0.23	1.47	0.315

* Statistically significant for p < 0.05.

## DISCUSSION

### Sample selection and methodology

The lack of a control group that would allow to separate growth changes effects from the MPA appliance effects, especially in Group 1, is a limitation of the present study. However, previous studies also were published with the absence of a control group, without prejudice of the results.^9^

The groups were compatible regarding sex distribution, malocclusion types (division 1 or 2, and bilateral or unilateral), and also regarding the orthodontists who conducted the treatments, which can influence the results (Table 1). Only the pre and posttreatment ages were significantly different between the groups, as expected, since the study compares groups with different age ranges (Tables 2 and 4).

### Maxillary skeletal component

Adults presented significantly greater maxillary retrusion (A-Nperp) at the pretreatment stage (Table 2). The effective length of the maxilla significantly increased in the adolescent group, which was expected because these patients were still in the growing stage of craniofacial development^20^ ^-^ ^22^ (Co-A; Table 4). Nevertheless, the therapy with the MPA did not seem to have influenced maxillary anterior displacement because the adult group continued to present significantly greater maxillary retrusion as compared to the adolescent group at the posttreatment stage (Table 3). Similar results have been previously observed.^9^ ^,^ ^23^

### Mandibular skeletal component

The adult group presented greater mandibular body (Go-Gn) and effective mandibular length (Co-Gn), as well as mandibular ramus height (Co-Go) at the pretreatment stage, and the adolescents had greater increase in these structures during the treatment period (Tables 2 and 4). The MPA may have contributed to some of this growth increase.^20-22^ On the other hand, growth changes in the adult group was negligible so that at the posttreatment stage the sizes of these mandibular structures were similar in the groups (Table 3). 

### Maxillomandibular relationship

At the pretreatment stage, the adolescent group presented a significantly greater apical base Class II discrepancy and profile convexity than the adult group (Table 2). The adolescents still presented a significantly greater Class II apical base discrepancy and convex facial profile than the adults at the posttreatment stage, although milder than at the pretreatment stage (Tables 3 and 4). Longitudinal comparisons indicate that growth trends are essentially similar between Class II division 1 and normal subjects in the various dentofacial parameters compared. The differences in mandibular length and position are more evident in the early stages of development than at the later stages. This may indicate the possibility of a “catch up” period in mandibular growth in Class II division 1 subjects at the later stages of development.^24^

### Vertical component

Both groups were very similar in the pretreatment stage regarding the growth pattern, with only the adult patients presenting a significantly greater posterior face height than the adolescents and therefore a more horizontal growth tendency (Table 2). However, as the adolescents had significantly greater increase in the lower anterior and posterior face heights, there were no significant differences in any variable of the vertical components at the posttreatment stage (Tables 3 and 4). Most likely these greater increases were consequent to growth in the adolescents because the adult group also experienced slight increases in these variables. These results would be expected, since they have been shown before.^25^

### Maxillary dentoalveolar component

As it would be expected, in the pretreatment stage the adults had significantly greater maxillary molar dentoalveolar height and mesial positioning than the adolescents, since growth had already been fully expressed (Table 2).^26^ During treatment, there was significantly greater palatal tipping of the maxillary incisors in the adolescents, most likely due to the appliance effects, and also greater maxillary molar vertical dentoalveolar development and mesialization, most probably due to growth.^22^ ^,^ ^26^ With these changes, the maxillary incisors ended up still with greater palatal tipping in the adolescents than in the adults and the maxillary molar dentoalveolar height became similar in the groups, in the posttreatment stage (Table 3).

### Mandibular dentoalveolar component

Similar to the maxillary molars, in the pretreatment stage the adults had significantly greater mandibular molars dentoalveolar height and mesial positioning than the adolescents, because growth had already been fully expressed (Table 2).^22^ ^,^ ^27^ During treatment, the adolescents had greater dentoalveolar development of the mandibular incisors and molars, probably due to the greater vertical development of the alveolar processes on growing patients, in comparison to adults (Table 4).^22^ Although the other dentoalveolar changes were not significantly different between the groups, the adult patients had a significantly greater proclination of the mandibular incisors at the posttreatment stage (Table 3). Probably this was the result of a cumulative effect of a greater non-significant mandibular incisor proclination at the pretreatment stage and during treatment that ultimately produced a significantly greater proclination at the posttreatment stage. Greater dentoalveolar changes may be expected in adult patients under treatment with fixed functional appliances because the skeletal changes are minimal.^7^ ^,^ ^9^ Besides, the adult patients had greater initial mandibular crowding which could have contributed to some incisor flaring during the initial stages of leveling and alignment.^3^ ^,^ ^28^

### Dental relationships

The overjet, overbite and molar relationship were similar in the groups at the pretreatment stage as were the treatment changes (Tables 2 and 4). Only the overbite was significantly smaller in the adult group at the posttreatment stage. Again this result may be the cumulative effect of a non-significantly greater overbite in the adolescent group in the pretreatment stage, associated to a non-significantly greater reduction in overbite in the adult group.

### Soft tissue component

The upper and lower lips were significantly more protruded in the adolescents than in the adults at the pretreatment stage (Table 2), in accordance with Pecora et al.^29^ During treatment the upper lip of the adolescents had significantly greater retraction than in the adult patients (Table 4). However, despite the greater retraction, the upper lip continued to exhibit greater protrusion in the adolescents, in the posttreatment period (Table 3).

## CLINICAL CONSIDERATIONS

The MPA is indicated in adult patients when they are not willing to cooperate with the use of removable appliances^30^ and also when they refuse to go through extractions or orthognathic surgery. Moreover, the MPA is an affordable option compared to other fixed devices to correct Class II malocclusions.^3^ ^,^ ^7^ ^,^ ^10^

The amount of skeletal changes was greater in the young group when compared to adult patients, nevertheless, it does not mean that the MPA causes more effects on growing subjects when compared to non-growing patients.^9^ Probably it was growth, and not the MPA, the responsible for these differences in the amount of skeletal changes between the two groups.^20^

At the posttreatment stage, no differences regarding mandibular size and position could be seen. This way, MPA has enough potential to warrant its use when indicated even in adult patients, since these dental effects are satisfactory to benefit Class II malocclusion correction.^9^ ^,^ ^31^

One has to bear in mind that the results of this study reflect the total treatment period including leveling and alignment, active retention and the finishing procedures, all performed with fixed appliances. Therefore, it should be emphasized that the changes of both groups are the results of the joint effects of the MPA and fixed appliances and not the MPA alone. On the other hand, this may be more informative than knowing only the changes produced by the MPA alone, because it provides information of the whole treatment. Patients usually are submitted to complete treatment.

Stability is always a concern with Class II malocclusion treatment with fixed functional appliances, especially in adults. Therefore, to increase stability of the anteroposterior correction obtained by the MPA after its removal, Class II elastics were used and their daily use was gradually reduced as stability was observed in each case. This is a usual active retention alternative employed after using a fixed functional appliance.^10^ ^,^ ^32^ Because use of the MPA appliance was of 9 months and treatment times were relatively long in both groups, the additional time with Class II elastics use as active retention should, most likely, assure good stability of the anteroposterior correction.^10^ ^,^ ^23^ Nevertheless, this issue should be investigated in future studies. 

## CONCLUSIONS

» Adult patients treated with MPA showed significant fewer amounts of skeletal, dentoalveolar and soft tissue changes than adolescents.

» Regarding appliance effects, there was significantly greater palatal tipping of maxillary incisors and retrusion of upper lip in the adolescents. Adults group showed greater mandibular incisor proclination in posttreatment stage.

## References

[1] Harris EF, Vaden JL, Dunn KL, Behrents RG (1994). Effects of patient age on postorthodontic stability in Class II, division 1 malocclusions. Am J Orthod Dentofacial Orthop.

[2] Mathews DP, Kokich VG (1997). Managing treatment for the orthodontic patient with periodontal problems. Semin Orthod.

[3] Gonner U, Ozkan V, Jahn E, Toll DE (2007). Effect of the MARA appliance on the position of the lower anteriors in children, adolescents and adults with Class II malocclusion. J Orofac Orthop.

[4] Kinzinger G, Diedrich P (2005). Skeletal effects in class II treatment with the functional mandibular advancer (FMA). J Orofac Orthop.

[5] Nalbantgil D, Arun T, Sayinsu K, Fulya I (2005). Skeletal, dental and soft-tissue changes induced by the Jasper Jumper appliance in late adolescence. Angle Orthod.

[6] Paulsen HU, Karle A (2000). Computer tomographic and radiographic changes in the temporomandibular joints of two young adults with occlusal asymmetry, treated with the Herbst appliance. Eur J Orthod.

[7] Ruf S, Pancherz H (2006). Herbst/multibracket appliance treatment of Class II division 1 malocclusions in early and late adulthood a prospective cephalometric study of consecutively treated subjects. Eur J Orthod.

[8] Ruf S, Pancherz H (1999). Temporomandibular joint remodeling in adolescents and young adults during Herbst treatment: a prospective longitudinal magnetic resonance imaging and cephalometric radiographic investigation. Am J Orthod Dentofacial Orthop.

[9] Pontes LF, Maia FA, Almeida MR, Flores-Mir C, Normando D (2017). Mandibular Protraction Appliance Effects in Class II Malocclusion in Children, Adolescents and Young Adults. Braz Dent J.

[10] Herrera FS, Henriques JF, Janson G, Francisconi MF, de Freitas KM (2011). Cephalometric evaluation in different phases of Jasper Jumper therapy. Am J Orthod Dentofacial Orthop.

[11] Furquim BD, Henriques JF, Janson G, Siqueira DF, Furquim LZ (2013). Effects of mandibular protraction appliance associated to fixed appliance in adults. Dental Press J Orthod.

[12] Johnston LE (2005). If wishes were horses functional appliances and growth modification. Progress Orthod.

[13] Coelho CM (1995). Mandibular protaction appliance for Class II treatment. J Clin Orthod.

[14] Pancherz H (1979). Treatment of class II malocclusions by jumping the bite with the Herbst appliance. A cephalometric investigation. Am J Orthod.

[15] Coelho CM (2002). Clinical application of the mandibular protraction appliance in upper lateral agenesy and in asymmetric cases. Tex Dent J.

[16] White LW, Coelho CM (2003). Treating Adults with the Mandibular Protraction Appliance.

[17] Stucki N, Ingervall B (1998). The use of the Jasper Jumper for the correction of Class II malocclusion in the young permanent dentition. Eur J Orthod.

[18] Dahlberg G (1940). Statistical methods for medical and biological students.

[19] Houston WJB (1983). The analysis of errors in orthodontic measurements. Am J Orthod.

[20] Behrents R (1985). Growth in the aging craniofacial skeleton.

[21] Bishara SE, Jamison JE, Peterson LC, DeKock WH (1981). Longitudinal changes in standing height and mandibular parameters between the ages of 8 and 17 years. Am J Orthod.

[22] Martins DR, Janson G, Henriques JFC, Freitas MR, Almeida RR (1998). Atlas de crescimento craniofacial.

[23] Siqueira DF, Almeira RR, Janson G, Brandao AG, Coelho CM (2007). Dentoskeletal and soft-tissue changes with cervical headgear and mandibular protraction appliance therapy in the treatment of Class II malocclusions. Am J Orthod Dentofacial Orthop.

[24] Bishara SE, Jakobsen JR, Vorhies B, Bayati P (1997). Changes in dentofacial structures in untreated Class II division 1 and normal subjects a longitudinal study. Angle Orthod.

[25] Stahl F, Baccetti T, Franchi L, McNamara JA (2008). Longitudinal growth changes in untreated subjects with Class II Division 1 malocclusion. Am J Orthod Dentofacial Orthop..

[26] Vaden JL, Harris EF, Behrents RG (1995). Adult versus adolescent Class II correction: a comparison. Am J Orthod Dentofacial Orthop.

[27] Konik M, Pancherz H, Hansen K (1997). The mechanism of Class II correction in late Herbst treatment. Am J Orthod Dentofacial Orthop.

[28] Richardson ME (1995). A preliminary report on lower arch crowding in the mature adult. Eur J Orthod.

[29] Pecora NG, Baccetti T, McNamara JA (2008). The aging craniofacial complex a longitudinal cephalometric study from late adolescence to late adulthood. Am J Orthod Dentofacial Orthop.

[30] Faltin KJ, Faltin RM, Baccetti T, Franchi L, Ghiozzi B, McNamara JA (2003). Long-term effectiveness and treatment timing for Bionator therapy. Angle Orthod.

[31] Coelho CM (2003). Entrevista. Rev Clín Ortod Dental Press.

[32] Sood S, Kharbanda OP, Duggal R, Sood M, Gulati S (2011). Neuromuscular adaptations with flexible fixed functional appliance--a 2-year follow-up study. J Orofac Orthop.

